# Commentary: Predictive value of serum miR-21 and miR-122 expression on the efficacy of capecitabine combined with transcatheter hepatic arterial embolization chemotherapy for liver metastasis after colorectal cancer surgery in patients with colorectal cancer and construction and verification of nomograms

**DOI:** 10.3389/fonc.2025.1668214

**Published:** 2025-10-01

**Authors:** Mengqi Ding, Nan Ling, Chaojun Wei, Jiangping Liu

**Affiliations:** ^1^ School of Public Health, Gansu University of Chinese Medicine, Lanzhou, China; ^2^ NHC Key Laboratory of Diagnosis and Therapy of Gastrointestinal Tumor, Gansu Provincial Hospital, Lanzhou, China; ^3^ Institute of Clinical Research and Translational Medicine, Gansu Provincial Hospital, Lanzhou, China

**Keywords:** colorectal cancer, liver metastases, transcatheter hepatic artery embolization chemotherapy, miR-21, miR-122, commentary

## 1 Introduction

In recent years, early diagnosis and treatment of cancer, particularly colorectal cancer, have been a significant focus in oncology research (1). Due to its high incidence and mortality rates, colorectal cancer (CRC) has become one of the major challenges in global cancer prevention and treatment (2). With advances in precision medicine, microRNAs (miRNAs) have garnered increasing attention as potential biomarkers for cancer diagnosis and therapy (3). Specifically, the expression patterns of miR-21 and miR-122 in colorectal cancer and their relationship with treatment outcomes have become a research hotspot (4). Ma et al. discussed the predictive value of miR-21 and miR-122 in patients with colorectal cancer liver metastasis treated with capecitabine and transcatheter arterial chemoembolization (TACE), and constructed a corresponding prognostic model (5). While the study is innovative and provides theoretical support for individualized treatment, its data analysis and model-building methods have limitations that may compromise the reliability and generalizability of the results. This manuscript examines these methods, focusing specifically on sample size, model validation, and potential overfitting. Furthermore, it analyzes challenges to the findings’ clinical application and proposes improvements to enhance the study’s overall robustness.

## 2 Commentary and discussion

This investigation into serum miR-21 and miR-122 expression as predictors of treatment efficacy in colorectal cancer patients with postoperative liver metastasis demonstrates exceptional scientific value and academic merit. The research addresses a critical clinical challenge in oncology by focusing on outcome prediction for colorectal cancer liver metastasis patients, directly responding to the evolving landscape of precision medicine and biomarker implementation. The investigators moved beyond examining miR-21 and miR-122 in isolation, instead pioneering an integrated approach that combines these microRNAs with established clinical indicators in a multivariable model for personalized treatment response prediction. This sophisticated integration strategy represents a significant advancement in tumor biomarker research methodology, creating a practical framework for assessing tumor heterogeneity and guiding individualized therapeutic interventions.

Methodological excellence characterizes both the experimental design and statistical analysis. The study population was carefully defined through comprehensive inclusion and exclusion criteria, ensuring cohort homogeneity and minimizing confounding factors. Treatment response assessment adhered rigorously to internationally standardized RECIST criteria, establishing a robust foundation for reproducibility. The molecular detection methodology employed precise reverse transcription-quantitative polymerase chain reaction (RT-qPCR) quantification protocols with meticulous documentation of sample handling procedures and implementation of technical replicates and quality control measures. These standardized approaches substantially enhanced data accuracy and reliability, strengthening the validity of the findings.

The data analysis framework demonstrates particular sophistication, utilizing logistic regression modeling complemented by a comprehensive performance evaluation strategy. The researchers employed multiple assessment methods including receiver operating characteristic (ROC) curve analysis for discrimination capacity, calibration curves to assess probability alignment, and decision curve analysis to evaluate clinical utility across threshold ranges. This thorough validation approach not only establishes statistical robustness but provides clinicians with accessible tools for practical implementation. The nomogram developed through this research exemplifies clinical translatability through its intuitive visualization design, facilitating potential adoption in practice settings. Furthermore, the study effectively bridges molecular mechanisms with clinical applications, elucidating both the tumor-promoting functions of miR-21 and the metastasis-inhibiting properties of miR-122, while contextualizing the predictive model’s relevance for post-surgical treatment decision-making.

Despite its analytical strengths and the clinical relevance of its topic, the study investigating serum miR-21 and miR-122 as predictors of postoperative therapeutic efficacy in patients with colorectal cancer liver metastasis exhibits notable shortcomings in key aspects of its research design and data analysis.

A primary limitation of the study is that the sample size and data partitioning strategy compromise its statistical power. A total of 252 patients were included and randomly allocated in a 7:3 ratio to a training set (*n* = 181) and a validation set (*n* = 71) without stratification by outcomes or key covariates. The validation set constituted only 28.2% of the cohort and contained fewer than 30 effective events (defined as “treatment ineffective” cases), which substantially diminished the statistical power (6). In the training set, the 86 effective events and 6 predefined variables yielded an event-per-variable (EPV) ratio of 14.3. This value, while marginally satisfying the conventional minimum requirement of EPV ≥ 10, is insufficient to robustly control for overfitting, particularly given the model’s inclusion of mixed variable types (7).

Furthermore, a single fixed data split was employed instead of more robust methods such as cross-validation or bootstrapping, thereby increasing the model’s susceptibility to random fluctuations (8). This limitation is reflected in the validation set performance, where the model’s area under the curve (AUC) declined from 0.810 in the training set to 0.731 in the validation set, a reduction of 0.079. The wide 95% confidence interval for the validation AUC (0.59-0.866), with a lower bound approaching 0.6, indicates that the model’s predictive performance may be only modestly superior to chance. Moreover, the validation set sensitivity was only 0.600, implying a 40% miss rate for ineffective treatment cases. This performance, combined with a negative likelihood ratio of 0.59, is suboptimal from both statistical and clinical standpoints. Additionally, increasing the sample size and employing stratified sampling for data partitioning would enhance the robustness of the model’s validation. Future studies should prioritize using cross-validation or bootstrap resampling techniques to improve the generalizability of the findings and mitigate the risks of overfitting.

The reporting of data integrity similarly lacks transparency. The methods section asserts that the final 252 included cases exhibited “no missing values,” yet it concurrently mentions the application of complete case analysis to a small number of missing cases. However, no details are provided regarding the original proportions of missing data, patterns of missingness (such as those assessed via Little’s test for missing completely at random assumptions), or attrition curves. In a retrospective cohort encompassing more than 15 indicators, including body mass index (BMI), carcinoembryonic antigen (CEA), carbohydrate antigen 19-9 (CA19-9), tumor size, and microvascular invasion, the complete absence of missing values is highly improbable and raises concerns about potential selection bias or reporting bias (9).Additionally, it is critical to discuss the implications of potential selection bias, as it is unlikely for key variables to have zero missing data in a retrospective cohort. A thorough assessment of missing data patterns and biases should be included to understand how these factors may influence the study’s findings.

The study also exhibits deficiencies in variable handling and model construction. The authors followed the conventional paradigm of univariate screening with a significance threshold of *P* < 0.05 for inclusion in the multivariate model, which may have prematurely excluded potentially important confounders or interacting variables, such as tumor differentiation and preoperative CA19–9 levels (10). Moreover, the assumption of linearity for all continuous variables is problematic, as it was justified solely by a statement that locally estimated scatterplot smoothing (LOESS) assessments revealed no evident nonlinearity, without accompanying graphical evidence or F-test statistics (11). Given the training set size of n=181, the application of a 3-knot restricted cubic spline would have been a feasible approach to accommodate potential nonlinear relationships between variables such as CEA or BMI and the outcome. Of greater concern, multicollinearity assessments, such as variance inflation factor (VIF) calculations, were entirely omitted despite the inclusion of correlated predictors (12). For example, the Spearman correlation coefficient between CEA and tumor size was 0.46, a level that could compromise the stability of regression coefficients and associated P-values. Finally, potential interactions among predictors were not evaluated, including those between miR-21 and miR-122, even though prior research indicates possible synergistic or antagonistic effects within the PI3K/AKT signaling pathway (13, 14). Addressing these concerns, it is crucial to apply robust statistical techniques to ensure that assumptions regarding linearity, multicollinearity, and variable interactions are thoroughly examined. Conducting sensitivity analyses and incorporating spline terms or transformations for continuous variables could provide a more accurate representation of the relationships between predictors and outcomes. Additionally, a systematic evaluation of potential interactions among predictors is warranted, especially in the context of known biological pathways.

The predictive performance evaluation also reveals substantial inadequacies in calibration, extending beyond the previously noted decline in AUC. In the validation set, the nomogram demonstrated a mean absolute calibration error of 0.210, reflecting a 21-percentage-point discrepancy between predicted probabilities and observed event rates, which indicates suboptimal calibration. Although the Hosmer-Lemeshow goodness-of-fit test produced a chi-squared value of 6.57 (*P* = 0.37), suggesting nominal adequacy, this metric is underpowered in smaller samples (*n* < 400) and therefore does not reliably substantiate effective calibration (15). Additionally, the authors omitted more robust indicators, such as the Brier score or calibration belt plots. The decision curve analysis (DCA) depicted net benefits across an extensive threshold probability range (0.05-0.95), presenting an implausibly favorable profile that is atypical for clinical prediction models and likely indicative of errors in plotting or computation. A theoretical recalculation of net benefits, incorporating the reported sensitivity of 0.600 and specificity of 0.793, would yield values nearing zero at thresholds exceeding 0.40.

Statistical inference in the study is compromised by unaddressed multiple comparisons and information leakage. Univariate analyses examined 18 candidate variables without implementing any multiplicity corrections, such as the Bonferroni or Benjamini-Hochberg procedures (16). At a significance level of α = 0.05, this approach would be expected to produce approximately 0.9 false positives, and with 7 variables identified as significant, the true false-positive rate remains indeterminate. Additionally, the thresholds for miR-21 (> 2.0) and miR-122 (< 0.5) were initially derived from prior literature and subsequently “validated” within the same dataset using ROC curve analysis with Youden indices (maximum values of 0.522 and 0.458, respectively). This process constitutes circular reasoning, which introduces information leakage and artificially inflates the AUC. Most critically, the interpretation of results exhibits a profound contradiction: the outcome was defined as treatment effectiveness (coded as 1) versus ineffectiveness (coded as 0), yet the multivariate regression table reports an odds ratio (OR) of 2.35 (95% CI 1.11-4.95, *P* = 0.025) for high miR-21 expression (coded as 1), which statistically implies an elevated probability of effectiveness. This discrepancy highlights potential issues in the coding and interpretation of variables within the model, suggesting that there may be underlying biases or confounding factors that have not been adequately addressed. Thus, it is vital for future research to thoroughly investigate these aspects to ensure clarity and consistency in the findings. This finding directly opposes the manuscript’s recurrent assertion that high miR-21 expression represents a risk factor for treatment ineffectiveness, thereby introducing a fundamental logical inconsistency that erodes the validity of the primary conclusions, potentially attributable to errors in coding or interpretation.

The reporting standards in the study do not meet the minimum requirements for reproducibility. Random seeds were not specified, and neither R code nor SPSS outputs were provided. Additionally, inconsistencies in variable labeling are evident: in the nomogram, “X2” is designated as CEA, whereas Table 2 identifies CEA as “X5,” which diminishes readability and complicates efforts to replicate the findings. Figure 3 presents ROC curves without axis scale markings, and essential details such as raw RT-qPCR cycle threshold (Ct) values, ΔCt calculation formulas, and efficiency correction parameters are entirely absent, thereby hindering peers from independently verifying the detection methodology. To improve reproducibility and transparency, it is crucial for authors to provide comprehensive information on random seeds, statistical codes, and outputs. Sharing this information not only facilitates verification but also enhances the credibility of the research. Furthermore, the authors do not outline specific interventions for patients classified as having “ineffective” treatment predictions or provide data on 1-year or 3-year overall survival rates, which restricts the model’s clinical applicability and long-term utility. The inclusion of these details would significantly enrich the research, allowing for better-informed decisions in clinical practice. To improve the clinical significance and applicability of future research, it is crucial to integrate survival data, which can provide valuable insights into patient outcomes over time. Linking clinical predictions with actionable measures is essential for guiding clinical practice effectively. Establishing a framework that combines predictive modeling with specific interventions can enable clinicians to make more informed decisions, ultimately enhancing patient management and care.

Despite these limitations, it is worth acknowledging that the study’s focus aligns well with the pressing clinical need for noninvasive and repeatable biomarkers for monitoring purposes. The integration of molecular biology insights with clinical decision-making represents a positive contribution to the field. Moreover, the research demonstrates a certain level of methodological rigor, ensuring the fundamental reliability of molecular data through the adoption of internationally recognized standards and standardized techniques. The study also exhibits innovation through its multi-component integration, patient stratification approaches, and standardized analytical workflows, which could offer novel strategies for the precision management of colorectal cancer metastasis and enhance clinical methods for evaluating treatment efficacy in patients with liver metastases. These exploratory endeavors provide an initial framework for optimizing personalized treatment regimens, facilitating multi-center applications, and directing subsequent mechanistic studies in oncology.

In summary, although the study by Ma et al. presents an innovative hypothesis with substantial clinical implications and undertakes preliminary investigations, its predictive model is undermined by a series of critical shortcomings in data analysis and methodology, including insufficient sample size, inappropriate modeling approaches, biased validation procedures, severe overfitting, and fundamental contradictions in the core conclusions. These issues collectively compromise the model’s reliability and validity in its present form, rendering it unsuitable for clinical guidance. To ensure that this valuable scientific inquiry can meaningfully advance the field, future studies should address several key improvements. First, researchers ought to increase sample sizes and implement multi-center designs, stratified sampling, or prospective cohorts, while substituting single data splits with cross-validation or nested bootstrapping to bolster model stability and generalizability. Second, modern penalized regression techniques, such as least absolute shrinkage and selection operator (LASSO) or elastic net, should be employed for integrated variable selection and multicollinearity management, alongside restricted cubic splines to account for potential nonlinear relationships in continuous variables and formal testing for interactions. Third, standardized practices for reporting and reproducibility must be adopted, encompassing the disclosure of analytical code, specification of random seeds, explicit description of missing data handling, and independent optimization of miRNA thresholds using external datasets to mitigate information leakage. Ultimately, integrating short-term efficacy predictions with validation against long-term survival endpoints, complemented by model-informed clinical decision-making trials, could enable this research avenue to yield more dependable and actionable tools for precision therapy in metastatic colorectal cancer.
